# S186. KCNH2 POLYMORPHISM ASSOCIATED TO ALTERED EEG FUNCTIONAL NETWORK MODULATION IN SCHIZOPHRENIA

**DOI:** 10.1093/schbul/sby018.973

**Published:** 2018-04-01

**Authors:** Alba Lubeiro Juárez, Vicente Molina Rodriguez, Maria Guardiola, Javier Gómez Pilar, Mar Fatjó-Vilas

**Affiliations:** 1 University of Valladolid; 2 FIDMAG Sisters Hospitallers Research Foundation; 3 University of Barcelona, Biomedicine Institute of the University of Barcelona (IBUB), Centre for Biomedical Research Network on Mental Health (CIBERSAM)

## Abstract

**Background:**

The rs3800779 polymorphism at KCNH2 gene, which encodes for a Voltage-Gated Potassium Channel Subunit, has been associated with the risk for schizophrenia (SZ) and with changes in the expression of a brain isoform with specific electrophysiological characteristics (Huffaker et al., 2009). It has been hypothesized that the KCNH2 gene variability could be involved in SZ by means of modulating the neuronal excitability. Graph-theory parameters applied to electroencephalographic (EEG) activity are useful to assess functional connectivity in the brain and have shown altered patterns of global connectivity in schizophrenia. We aimed to investigate whether KCNH2 contributes to functional connectivity alterations replicated in SZ patients.

**Methods:**

EEG data were acquired during the performance of an odd-ball task in 50 schizophrenia patients and 101 matched healthy controls. The rs3800779 at KCNH2 was genotyped. From the EEG activity, the Small World index (SW, a measure of network efficiency) was calculated as the coefficient between clustering coefficient (CLC, a measure of network segregation) and path length (PL, a measure of network integration). SW was calculated in two temporal windows with respect to the target tone (pre-stimulus and response). Functional SW modulation (SWm) was calculated as the difference in SW between pre-stimulus and response windows. Finally, the association between KCNH2 polymorphism and functional connectivity modulation was assessed.

**Results:**

Patients carrying the A allele (AA or AC, n=25) showed smaller SW modulation in comparison with patients with the CC genotype (n=25) (t=-2.84, df=48, p=0.007). Moreover, patients carrying the A allele showed smaller SW modulation than healthy controls with the A allele (n=45) (t=-3.41, df=68, p=0.001) or without the A allele (n=56) (t=-3.87, df=79 p<0.001). There were no significant SW modulation differences between healthy controls carrying or not the A allele. Patients with the AA/AC genotype showed an inverse SW modulation pattern (decreased SW at response) in comparison with patients without the A allele and controls (increased SW at response).

**Discussion:**

Our data indicate that, within SZ patients, the A allele is associated with smaller SW modulation and lower SW values at response, which might be interpreted as an altered ability to coordinate the activity of neural assemblies during cognitive tasks (Basar et al 2016). Although replication analyses are needed, our findings suggest that genetic variation at KCNH2 might contribute to the efficiency of brain functional networks in schizophrenia patients.

**References:**

1. Basar, E., Golbasi, B.T., Tulay, E., Aydin, S., Basar-Eroglu, C., 2016. Best method for analysis of brain oscillations in healthy subjects and neuropsychiatric diseases. Int J Psychophysiol 103, 22–42.

2. \Huffaker, S.J., Chen, J., Nicodemus, K.K., Sambataro, F., Yang, F., Mattay, V., Lipska, B.K., Hyde, T.M., Song, J., Rujescu, D., Giegling, I., Mayilyan, K., Proust, M.J., Soghoyan, A., Caforio, G., Callicott, J.H., Bertolino, A., Meyer-Lindenberg, A., Chang, J., Ji, Y., Egan, M.F., Goldberg, T.E., Kleinman, J.E., Lu, B., Weinberger, D.R., 2009. A primate-specific, brain isoform of KCNH2 affects cortical physiology, cognition, neuronal repolarization and risk of schizophrenia. Nature medicine 15(5), 509–518.

